# Life-Span Exposure to Low Doses of Aspartame Beginning during Prenatal Life Increases Cancer Effects in Rats

**DOI:** 10.1289/ehp.10271

**Published:** 2007-06-13

**Authors:** Morando Soffritti, Fiorella Belpoggi, Eva Tibaldi, Davide Degli Esposti, Michelina Lauriola

**Affiliations:** Cesare Maltoni Cancer Research Center, European Ramazzini Foundation of Oncology and Environmental Sciences, Bologna, Italy

**Keywords:** artificial sweeteners, aspartame, carcinogenicity, lymphomas/leukemias, mammary cancers, prenatal exposure, Sprague-Dawley

## Abstract

**Background:**

In a previous study conducted at the Cesare Maltoni Cancer Research Center of the European Ramazzini Foundation (CMCRC/ERF), we demonstrated for the first time that aspartame (APM) is a multipotent carcinogenic agent when various doses are administered with feed to Sprague-Dawley rats from 8 weeks of age throughout the life span.

**Objective:**

The aim of this second study is to better quantify the carcinogenic risk of APM, beginning treatment during fetal life.

**Methods:**

We studied groups of 70–95 male and female Sprague-Dawley rats administered APM (2,000, 400, or 0 ppm) with feed from the 12th day of fetal life until natural death.

**Results:**

Our results show *a*) a significant dose-related increase of malignant tumor–bearing animals in males (*p* < 0.01), particularly in the group treated with 2,000 ppm APM (*p* < 0.01); *b*) a significant increase in incidence of lymphomas/leukemias in males treated with 2,000 ppm (*p* < 0.05) and a significant dose-related increase in incidence of lymphomas/leukemias in females (*p* < 0.01), particularly in the 2,000-ppm group (*p* < 0.01); and *c*) a significant dose-related increase in incidence of mammary cancer in females (*p* < 0.05), particularly in the 2,000-ppm group (*p* < 0.05).

**Conclusions:**

The results of this carcinogenicity bioassay confirm and reinforce the first experimental demonstration of APM’s multipotential carcinogenicity at a dose level close to the acceptable daily intake for humans. Furthermore, the study demonstrates that when life-span exposure to APM begins during fetal life, its carcinogenic effects are increased.

Aspartame (APM) is one of the most widely used artificial sweeteners in the world. First approved by the U.S. Food and Drug Administration (FDA) for limited use in solid food in 1981, its authorization was extended to soft drinks in 1983 and then approved as a general sweetener in 1996 (FDA 1981, 1983, 1996). Likewise, the sweetener was approved for general use in the European Union in 1994 (EC Directive 1994). APM is now present in > 6,000 consumer packaged goods and in nearly 500 pharmaceutical products, including children’s medicines (Aspartame Information Center 2005). In the United States, > 70% of aspartame sales are attributed to soft drinks (American Dietetic Association 2004). The acceptable daily intake (ADI) of aspartame is currently 50 mg/kg body weight (bw) in the United States and 40 mg/kg bw in the European Union for both children and adults. Daily consumption of artificial sweeteners by women of childbearing age and by children has been estimated at 2.5–5.0 mg/kg bw (Butchko et al. 2002). In a study of Swedish diabetics, the general APM intake was lower than the ADI, but the worst-case calculation of intake in the children’s group was 114% of the ADI (Ilbäck et al. 2003).

APM is metabolized in the gastric tract of rodents, nonhuman primates, and humans to its three constituents: aspartic acid, phenylalanine, and methanol. When absorbed, aspartic acid is transformed into alanine plus oxaloacetate (Stegink 1984); phenylalanine is transformed mainly into tyrosine and, to a lesser extent, phenylethylamine and phenyl-pyruvate (Harper 1984); and methanol is transformed into formaldehyde and then to formic acid (Opperman 1984).

*In vitro* and *in vivo* tests demonstrate that APM is not genotoxic. Likewise, long-term carcinogenicity studies conducted by the manufacturers of aspartame using rats and mice in the 1970s and 1980s did not demonstrate any carcinogenic effects. A detailed review of the genotoxicity and carcinogenicity studies available to date on APM has been published previously (Belpoggi et al. 2006; Soffritti et al. 2005, 2006). In our opinion, the small number of animals used per sex and per group and the termination of these experiments after 110 weeks of age, rather than observing animals over their life span, represent limiting factors when evaluating the carcinogenic risk or safety of artificial sweeteners such as aspartame. It was for this reason, together with the growing use of APM in industrialized countries, that we designed and performed a mega-experiment using seven groups of Sprague-Dawley rats (100–150 per sex per group) treated with APM in feed at various dose levels (including one very close to the ADI for humans), from 8 weeks of age until natural death (Belpoggi et al. 2006; Soffritti et al. 2005, 2006). The study demonstrated for the first time that APM is a multipotential carcinogenic agent, capable of inducing, in our experimental conditions *a*) a significant, dose-related increased incidence of malignant tumor–bearing animals in males (*p* ≤ 0.05) and in females (*p* ≤ 0.01), particularly in females treated at 50,000 ppm (*p* ≤ 0.01); *b*) a significant dose-related increase in lymphomas/leukemias in both males (*p* ≤ 0.05) and females (*p* ≤ 0.01), particularly in females treated at doses of 100,000 (*p* ≤ 0.01), 50,000 (*p* ≤ 0.01), 10,000 (*p* ≤ 0.05), 2,000 (*p* ≤ 0.05), or 400 ppm (*p* ≤ 0.01); *c*) a significant, dose-related increased incidence (*p* ≤ 0.01) of transitional cell carcinomas of the renal pelvis and ureter and their precursors (dysplasias) in females treated at 100,000 (*p* ≤ 0.01), 50,000 (*p* ≤ 0.01), 10,000 (*p* ≤ 0.01), 2,000 (*p* ≤ 0.05), or 400 ppm (*p* ≤ 0.05); *d* ) a significant, dose-related increased incidence of malignant schwannomas of peripheral nerves (*p* ≤ 0.05) in males (Belpoggi et al. 2006; Soffritti et al. 2005, 2006).

Given the consolidated experience of the European Ramazzini Foundation (ERF) in the conduct of long-term bioassays and the large number of rodents used in the study, the results attracted the attention of the scientific community, consumer and industry associations, and the national and international agencies responsible for food safety, including the Italian Superior Council of Health, the European Food Safety Authority (EFSA), the U.S. FDA, Health Canada, and others. At their request, we provided each of these agencies with all available raw data related to the study.

To our knowledge, only the EFSA has issued an official opinion on our study, releasing on 5 May 2006 a 40-page report in which they concluded that it is not necessary to revise their previous opinion on the absolute safety of APM (EFSA 2006).

Subsequent to our findings of hematopoietic cancers in rats, and in light of persistent concerns among the scientific community of an association between APM and brain cancers, Lim et al. (2006) published the results of a study that assessed the correlation between the consumption of aspartame-containing beverages and the incidence of these types of cancers. The findings were based on data derived from a prospective study conducted by the U.S. National Institutes of Health and the American Association of Retired Persons, using a cohort of > 285,000 men and > 188,000 women between 50 and 71 years of age, who had satisfactorily responded to a self-administered food frequency questionnaire. The questionnaire included questions on the consumption of beverages (soft drinks, fruit drinks, sweetened iced tea) potentially containing APM during the previous year. The questionnaires were mailed from 1995 to 1996 and the follow-up lasted until 2000. The conclusions of the study (Lim et al. 2006) did not support the hypothesis that APM increases hematopoietic or brain cancer risks.

Recently a group of Italian authors (Gallus et al. 2007) published the results of an integrated network of case–control studies conducted in Italy between 1991 and 2004 on the potential correlation between artificial sweeteners (including APM) and cancer. The authors interviewed patients with histologically confirmed cancers of the oral cavity and pharynx (598), esophagus (304), colon (1,225), rectum (728), larynx (460), breast (2,569), ovary (1,031), prostate (1,294), and kidney (renal cell carcinoma 767). Controls were 7,028 patients (3,301 men and 3,727 women) admitted to the same hospitals for acute, nonneoplastic disorders. Cases and controls were interviewed during their hospital stay, using a questionnaire on subjects’ usual diet in the 2 years before diagnosis. The results reported a lack of association between artificial sweeteners and the risk of the aforementioned cancers.

As soon as we perceived the carcinogenic effects of APM during the elaboration of the data in our first mega-experiment (Belpoggi et al. 2006; Soffritti et al. 2005, 2006), we planned an integrated program of long-term bioassays, beginning treatment from prenatal life, on > 4,000 rats and mice in order to better quantify the carcinogenic risks of aspartame. In this report we present the results of a second study on APM in which male and female Sprague-Dawley rats were exposed to very low doses of APM in feed (100 or 20 mg/kg bw) from fetal life until natural death.

## Materials and Methods

The APM used in this study was produced by Ajinomoto (Gravelines, France) and supplied by Giusto Faravelli S.p.A. (Milan, Italy). The purity of the APM, as determined by an infrared absorption spectrophotometer assay, was > 98.7%: diketopiperazine was < 0.3% and l-phenylalanine was < 0.5%. APM was added to the standard diet, which has been used for > 30 years at the Cesare Maltoni Cancer Research Center (CMCRC)/ERF, at APM concentrations of 2,000, 400, or 0 ppm to simulate an assumed daily APM intake of 100, 20, or 0 mg/kg bw. The feed was supplied by the producer on a monthly basis. The stability of the aspartame in feed was analyzed before the start of the study and periodically confirmed throughout the course of the biophase. The daily APM consumption (milligrams per kilogram body weight) was calculated estimating the average body weight for both males and females as 400 g for the duration of the experiment and the daily consumption of feed as 20 g/day.

The feed was supplied *ad libitum* to groups of 70–95 male and female Sprague-Dawley rats from the colony of the CMCRC/ERF. The basic tumorigram of this strain of rats is well known. Treatment began during fetal life, with administration of APM in feed to female breeders from the 12th day of pregnancy, when organogenesis is completed and before which time many tissues and organs are refractory to the effects of carcinogenic agents [International Agency for Research on Cancer (IARC) 1973]. The breeders were sacrificed after weaning, and treatment of the offspring lasted until natural death. Control animals received the same feed without APM.

At 4–5 weeks of age (after weaning), the experimental animals were identified by ear punch, separated by sex, and assigned to a respective dose group, depending on the APM concentration administered to the breeder. They were then housed five per cage in poly-carbonate cages (41 × 25 × 15 cm) with stainless-steel wire tops and a shallow layer of white wood shavings as bedding, and kept in a room used only for this experiment. The room was maintained at a temperature of 23 ± 2°C and relative humidity of 50–60%.

All animals were kept under observation until natural death. The experiment was conducted according to Italian law regulating the use and humane treatment of animals for scientific purposes (Decreto Legislativo N. 116 1992).

Mean daily drinking water and feed consumption were measured per cage, and body weight was measured individually, beginning at 6 weeks of age and continuing once each week for the first 13 weeks, then every 2 weeks until animals reached 110 weeks of age. Measurement of body weight continued every 2 weeks until the end of the experiment. To detect and register all gross lesions, the animals were clinically examined every 2 weeks for the duration of the experiment. To evaluate the status and behavior of the animals and to limit the postmortem modifications, a patrol was performed three times daily Monday–Friday and twice on Saturdays, Sundays, and holidays. Deceased animals were registered and kept refrigerated for a maximum of 16–19 hr at 4°C until necropsy.

The biophase ended at 147 weeks with the death of the last animal at the age of 144 weeks. Upon death, all animals underwent complete necropsy. Histopathology was routinely performed on the following organs and tissues of each animal from each group: skin and subcutaneous tissue, mammary gland, the brain (three sagittal sections), pituitary gland, Zymbal glands, salivary glands, Harderian glands, cranium (five sections, with oral and nasal cavities and external and internal ear ducts), tongue, thyroid, parathyroid, pharynx, larynx, thymus and mediastinal lymph nodes, trachea, lung and mainstem bronchi, heart, diaphragm, liver, spleen, pancreas, kidneys, adrenal glands, esophagus, stomach (fore and glandular), intestine (four levels), urinary bladder, prostate (male only), vagina (female only), gonads, interscapular brown fat pad, subcutaneous and mesenteric lymph nodes, and other organs or tissues with pathologic lesions. All organs and tissues were preserved in 70% ethyl alcohol except for bones, which were fixed in 10% formalin and then decalcified with 10% formaldehyde and 20% formic acid in water solution. The normal specimens were trimmed following CMCRC/ERF laboratory standard operating procedures. The pathologic tissue was trimmed to allow for the largest surface, including normal adjacent tissue. Trimmed specimens were processed in paraffin, and 3- to 5-μm sections of every specimen were obtained.

Sections were routinely stained with hematoxylin and eosin. All slides were examined microscopically by the same group of pathologists following the same criteria of histopathologic evaluation and classification. A senior pathologist reviewed all tumors and all other lesions of oncologic interest.

We performed statistical evaluations of the incidence and dose–response relationship of neoplastic lesions using the Cox regression model (Cox 1972). *p*-Values are reported in the tables.

## Results

**Figure 1:**
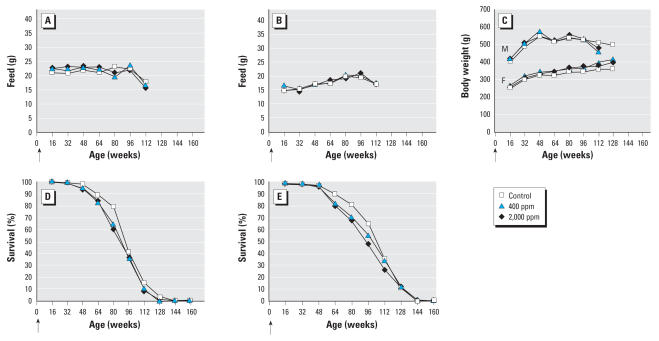
Effects of APM exposure on feed consumption, body weight, and survival. Mean daily feed consumption in males (*A*) and females (*B*). (*C*) Mean body weights in males (M) and females (F). Survival in males (*D*) and females (*E*). Arrows indicate the start of the experiment.

The experiment proceeded smoothly without unexpected occurrences. We observed no relevant differences in feed consumption between treated and untreated groups, in either males or females (Figure 1A,1B); no differences were observed in water consumption between groups or between sexes. No difference in mean body weight was observed in the treated groups compared with the controls (Figure 1C). We observed a slight decrease, seemingly dose related, in survival in the treated groups compared with the control group in both males and females (Figure 1D,1E).

Oncologic results are reported in Tables 1 and 2 for males and females, respectively. Multiple tumors of different type and site, of different type in the same site, of the same type in bilateral organs, of the same type in the skin, in subcutaneous tissue, in mammary glands, or at distant sites of diffuse tissue (i.e., bones and skeletal muscle) were plotted as single/independent tumors. Multiple tumors of the same type in the same tissue and organ, apart those listed above, were plotted only once.

### Total malignant tumors

The incidence of malignant tumor–bearing animals occurred with a significant, dose-related increase in males (*p* ≤ 0.01). The incidence of malignant tumors was significantly increased in males treated with 2,000 ppm APM (*p* ≤ 0.01) compared with controls (Table 1). Albeit not significant, a numeric increase of the incidence of animals bearing malignant tumors was also observed among females exposed to 2,000 ppm APM compared with controls (Table 2). Tumor types that contributed most to this increased incidence are presented below.

### Lymphomas/leukemias

The occurrence of lymphomas/leukemias in males and females is reported in Tables 1 and 2. The data show that APM causes a significant, dose-related increased incidence in females (*p* ≤ 0.01). When compared with the untreated control group, the increased incidence of lymphomas/ leukemias in treated males and females was significant at 2,000 ppm APM (*p* ≤ 0.05 and *p* ≤ 0.01, respectively). In males, the most frequent histotypes observed were lymphoimmunoblastic lymphomas that mainly involved lung and mediastinal/peripheral nodes. In females, the most frequent histotypes were lymphocitic lymphomas and lymphoimmunoblastic lymphomas that mainly involved the thymus, lung, spleen, and peripheral nodes. The differential diagnoses were based on the morphologic criteria regularly used in our laboratories, according to the guidelines of the International Classification of Rodent Tumors (IARC 1993). Lymphomas/leukemias (this term includes all types of hemolymphosarcomas and leukemias) are neoplasias arising from hemolymphoreticular tissues. Their aggregation is regularly used in experimental carcino-genesis because both solid and circulating phases are present in many lymphoid neoplasms, and distinction between them is artificial (Harris et al. 2001).

### Mammary carcinomas

The incidence of mammary gland carcinomas in males and females is reported in Tables 1 and 2. A dose-related increase in the incidence of carcinomas was observed in females (*p* ≤ 0.05). The incidence of lesions in females exposed to 2,000 ppm APM was significantly higher (*p* ≤ 0.05) compared with the controls. Two carcinomas were also observed among males treated with 2,000 ppm APM.

### Historical controls

In our laboratory over the last 20 years, the overall incidence of lymphomas/leukemias was 20.6% (range, 8.0–30.9%) among 2,265 male rats and 13.3% (range, 4.0–25.0%) among 2,274 female rats. The overall incidence of mammary cancers in the same group of female rats was 9.2% (range, 4.0–14.2%).

## Discussion

In our first mega-experiment (Belpoggi et al. 2006; Soffritti et al. 2005, 2006), we demonstrated for the first time that APM is a multi-potential carcinogenic agent inducing, among other cancers, a dose-related, significant increase in lymphomas/leukemias in females.

In the present study, in which we administered APM (2,000 and 400 ppm; equivalent to consumption of 100 and 20 mg/kg bw, respectively) to Sprague-Dawley rats in feed beginning during fetal life, we again confirmed that APM induces carcinogenic effects; we found *a*) a significant dose-related increase of malignant tumor–bearing animals in males (*p* < 0.01), in particular in the group treated with 2,000 ppm APM (*p* < 0.01); *b*) a significant increase in the incidence of lymphomas/ leukemias in males in the 2,000-ppm group (*p* < 0.05) and a significant dose-related increase in the incidence of lymphomas/ leukemias in females (*p* < 0.01), in particular in the 2,000-ppm group (*p* < 0.01); *c*) a significant dose-related increase in the incidence of mammary cancer in females (*p* < 0.05), particularly in the 2,000-ppm group (*p* < 0.05).

**Figure 2:**
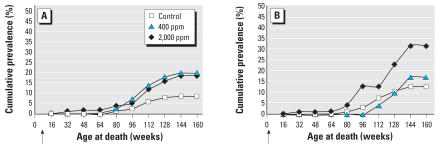
Cumulative prevalence of death by age in female rats bearing hemolymphoreticular neoplasias. (*A*) Postnatal APM exposure. (*B*) Prenatal APM exposure. Arrows indicate the start of the experiment.

When comparing life-span exposure beginning during prenatal and postnatal life, we have shown that prenatal exposure to APM clearly increases the incidence of lymphomas/leukemias in females (Table 3). Moreover, when comparing the cumulative prevalence by age of death of animals with hemolymphoreticular neoplasias, it is clear that prenatal exposure to APM also accelerates the insurgence of these lesions in females (Figure 2).

With regard to males, the incidence of lymphomas/leukemias in the concurrent control (9.5%) falls within the lower range of our historical controls (8.0–30.9%), and the incidence of lymphomas/leukemias in the group treated at the highest dose (17.1%) is close to the overall historical incidence (20.9%). Because the incidence of lymphomas/leukemias observed in males treated with 2,000 ppm APM is close to double the concurrent control, we consider these effects to be related to APM exposure (Haseman 1992, 1995; Haseman et al. 1984).

The results of our second experiment (the present study) further disprove the alternative hypothesis suggested by the EFSA (2006) regarding the cause of lymphomas/leukemias in our colony, in which they considered the incidence of lymphomas/leukemias observed in our first experiment to be “unrelated to APM given the high background incidence of chronic inflammatory changes in the lung.” First, as previously reported (Soffritti 2006), experimental animals that are allowed to die spontaneously are subject to infectious pathologies that are part of the natural dying process in both rodents and humans. Second, among the animals bearing lymphomas/ leukemias, we observed the diffusion of neoplastic tissue not only in the lung but also concurrently in various organs (liver, spleen, mediastinal and other lymph nodes). Finally, it should be noted that out of 49 agents reported to be carcinogenic in rats by the CMCRC/ERF, only 8 of these agents induced hemolymphoreticular malignancies. Of these, 3 were demonstrated in both males and females—formaldehyde (Soffritti et al. 2002b), mancozeb (Belpoggi et al. 2002a), and di-isopropyl-ether (Belpoggi et al. 2002b)—and 5 only in females—toluene (Soffritti et al. 2004), methyl alcohol (Soffritti et al. 2002a), methyl *tert*-butyl ether (Belpoggi et al. 1995), *tert*-amyl-methyl-ether (Belpoggi et al. 2002b), and APM (Belpoggi et al. 2006; Soffritti et al. 2005, 2006).

The two aforementioned epidemiologic studies (Gallus et al. 2006; Lim et al. 2006) published after our first mega-experiment (Belpoggi et al. 2006; Soffritti et al. 2005, 2006) merit general comment. Both studies consider the eating habits of a large population of males and females 50–70 years of age in the 1990s. Given the time frame of these surveys and the commercialization of aspartame in the 1980s, the subjects’ potential use of the sweetener could not have exceeded 10–15 years. It is difficult to believe that this limited adult period of exposure to APM could confirm or exclude a potential carcinogenic risk. The design of these studies underlines the importance of conducting an epidemiologic study in which exposure to APM is monitored beginning in fetal life, particularly given the use of products containing APM by children and women of child-bearing age.

## Conclusions

The results of this study, our second long-term carcinogenicity bioassay on APM, not only confirm but also reinforce our first experimental demonstration (Belpoggi et al. 2006; Soffritti et al. 2005, 2006) of APM’s multipotental carcinogenicity at a dose level close to the human ADI. Furthermore, the study demonstrates that when life-span exposure to APM begins during fetal life, its carcinogenic effects are increased.

On the basis of the present findings, we believe that a review of the current regulations governing the use of aspartame cannot be delayed. This review is particularly urgent with regard to aspartame-containing beverages, which are heavily consumed by children.

## Correction

In the original manuscript published online, Michelina Lauriola’s name was spelled incorrectly. It has been corrected here.

## Figures and Tables

**Table 1:** Incidence of malignant tumors in male Sprague-Dawley rats exposed to APM from fetal day 12 throughout the life span.

		Malignant tumors^a^							
		Tumor-bearing animals^b^		Total tumors		Total animals bearing lymphomas/leukemias^c^		Total animals bearing mammary carcinomas	
APM dose, ppm (mg/kg bw)	No. of animals at start	No.	Percent	No.	No./100 animals	No.	Percent	No.	Percent
2,000 (100)	70	28	40.0**	31	44.3	12	17.1*	2	2.9
400 (20)	70	18	25.7	19	27.1	11	15.7	0	—
0 (0)	95	23	24.2**	26	27.4	9	9.5	0	—

a Tumor rates are based on the number of animals examined (necropsied).

b *p*-Value associated with the dose–response test is near the control incidence.

c In male historical controls (2,265 rats), the overall incidence of lymphomas/leukemias is 20.6% (range, 8.0–30.9%).

* Significant (*p* ≤ 0.05) using Cox regression model.

** Significant (*p* ≤ 0.01) using Cox regression model.

**Table 2:** Incidence of malignant tumors in female Sprague-Dawley rats exposed to APM from fetal day 12 throughout the life span.

		Malignant tumors^a^							
		Tumor-bearing animals		Total tumors		Total animals bearing lymphomas/leukemias^b^,^c^		Total animals bearing mammary carcinomas^c^	
APM dose, ppm (mg/kg bw)	No. of animals at start	No.	Percent	No.	No./100 animals	No.	Percent	No.	Percent
2,000 (100)	70	37	52.9	60	85.7	22	31.4**	11 (15)^d^	15.7*
400 (20)	70	31	44.3	44	62.9	12	17.1	5 (6)	7.1
0 (0)	95	42	44.2	48	50.5	12	12.6**	5 (6)	5.3*

a Tumor rates are based on the number of animals examined (necropsied).

b In female historical controls (2,274 rats), the overall incidence of lymphomas/leukemias is 13.3% (range, 4.0–25.0%), and of mammary cancers is 9.2% (range, 4.0–14.2%).

c *p*-Values associated with the dose–response test are near the control incidence.

d Number of animals (number of tumors); an animal can bear multiple tumors.

* Significant (*p* ≤ 0.05) using Cox regression model.

** Significant (*p* ≤ 0.01) using Cox regression model.

**Table 3:** Comparison of the incidence of lymphomas/leukemias in female Sprague-Dawley rats beginning APM exposure from postnatal or prenatal life.

	Percent of animals bearing lymphomas/leukemias	
APM dose, ppm (mg/kg bw)	Postnatal exposure^a^ (No. of animals at start)	Prenatal exposure (No. of animals at start)
2,000 (100)	18.7 (150)	31.4 (70)
400 (20)	20.0 (150)	17.1 (70)
0 (0)	8.7 (150)	12.6 (95)

a Data from Soffritti et al. (2006).
